# Retraction Note: One-step Conjugation of Glycyrrhetinic Acid to Cationic Polymers for High-performance Gene Delivery to Cultured Liver Cell

**DOI:** 10.1038/s41598-023-50131-2

**Published:** 2024-01-03

**Authors:** Yue Cong, Bingyang Shi, Yiqing Lu, Shihui Wen, Roger Chung, Dayong Jin

**Affiliations:** 1 Institute of Pharmacy, Pharmaceutical College, Henan University, Jin Ming Avenue, Kaifeng, 475004 Henan China; 2 College of Life Sciences, Henan University, Jin Ming Avenue, Kaifeng, 475004 Henan China; 3 Advanced Cytometry Labs, ARC Centre of Excellence for Nanoscale BioPhotonics (CNBP), Macquarie University, Sydney, NSW 2109 Australia; 4 Faculty of Medicine & Health Sciences, Macquarie University, Sydney, NSW 2109 Australia

Retraction of: *Scientific Reports* 10.1038/srep21891, published online 23 February 2016

Editors have retracted this Article.

After publication of the Author Correction to this Article, further concerns were raised about the corrected content. The Authors are unable to provide the original data underlying some of these experiments and requested the retraction. Therefore, Editors no longer have confidence in the reliability of the data and the conclusions of this study.

All Authors agree with the retraction and its wording.

